# An immunoregenerative approach to mitigate post-traumatic osteoarthritis after intra-articular fracture

**DOI:** 10.1302/2046-3758.153.BJR-2024-0223.R3

**Published:** 2026-03-17

**Authors:** Michael S. Valerio, Jorge B. Edwards, Andrew R. Clark, Jessica M. Motherwell, Benjamin K. Potter, Christopher L. Dearth, Stephen M. Goldman

**Affiliations:** 1 Extremity Trauma and Amputation Center of Excellence, Defense Health Agency, Falls Church, Virginia, USA; 2 Department of Surgery, Uniformed Services University of the Health Sciences, Bethesda, Maryland, USA; 3 The Henry M. Jackson Foundation for the Advancement of Military Medicine, Inc., Bethesda, Maryland, USA; 4 Department of Orthopedic Surgery, Walter Reed National Military Medical Center, Bethesda, Maryland, USA

**Keywords:** Wounds and injuries, Inflammation, Osteochondral repair, post-traumatic osteoarthritis, post-traumatic osteoarthritis, Intra-articular fractures, Saline, staining, Cartilage, inflammation, anakinra, Serum, synovial fluid

## Abstract

**Aims:**

This study aimed to assess the effects of chondrogenic agents kartogenin (KGN) and KA9 in combination with anakinra (ANR), an interleukin (IL) 1 receptor antagonist (IL1RA) on post-traumatic osteoarthritis (PTOA) progression following intra-articular fractures (IAF). The hypothesis was that this immunoregenerative approach would promote osteochondral healing and alleviate PTOA more effectively than individual treatments or controls.

**Methods:**

Male Lewis rats (n = 78) underwent IAF, followed by weekly intra-articular injection of saline (control), KGN, or KA9 in conjunction with the systemic administration of saline (control) or ANR via osmotic pumps. Inflammatory and osteochondral markers were assessed through serum and synovial fluid analysis, micro-CT (µCT) for bone parameters, contrast-enhanced µCT for cartilage evaluation, histopathological analysis, and immunohistochemistry (IHC).

**Results:**

Immunoregenerative treatments did not adversely affect animal wellbeing. While local inflammation markers were minimally affected, some osteochondral remodelling markers indicated that KGN, KA9, and their combination with ANR reduced markers of osteochondral degradation. Bone analysis showed improved trabecular morphometry parameters and reduced bone surface with some treatments, and cartilage composition and thickness improved with treatments, although the effects were inconsistent across groups. Osteoarthritis Research Society International scores revealed location-specific variations in pathology, and IHC staining demonstrated intergroup differences in protein expression at different timepoints.

**Conclusion:**

This study suggests that KGN and KA9 may offer some protection against PTOA by influencing cartilage and subchondral bone health, and that the combination with ANR shows potential for enhanced effects. However, the results were mixed, with no treatment group consistently demonstrating positive improvements across all outcomes. The complexity of developing a novel immunoregenerative therapy for PTOA is highlighted by the variability in treatment response.

Cite this article: *Bone Joint Res* 2026;15(3):264–277.

## Article focus

- Intra-articular fractures increase the risk of degenerative post-traumatic osteoarthritis (PTOA), a condition characterized by rapid osteochondral deterioration.
- PTOA development is driven by tissue damage, joint incongruities, and dysregulated inflammation.
- A multi-pronged approach, combining anti-inflammatory therapies with chondrogenic agents, may enhance healing and reduce PTOA development after intra-articular fracture.

## Key messages

- Chondrogenic agents (kartogenin and KA9) demonstrate some potential to protect against PTOA development, but their effects, individually and in combination with an anti-inflammatory agent (anakinra), are inconsistent.
- Kartogenin and KA9, as well as their combination with ANR, showed some improvements in cartilage thickness and sulphated glycosaminoglycan density in certain instances.
- While the study’s findings are promising, they also highly the complexity of immunoregenerative therapy. The authors acknowledge the need for further research in alternative PTOA models, including higher-order species, to optimize treatment strategies and better define which specific aspects of PTOA progression these treatments can effectively address.

## Strengths and limitations

- The study employs a controlled experimental design with a saline control group and multiple treatment groups, utilizing a range of evaluation methods including CE-µCT, histological analysis, and biomarker assessment to comprehensively evaluate the treatment’s effects on cartilage thickness, composition, and osteochondral remodelling.
- While the study’s findings show promise, additional research is necessary to elucidate the mechanisms of immunoregenerative treatment in diverse PTOA models and species, ultimately informing optimized treatment protocols and enhancing clinical applicability.

## Introduction

Traumatic events, like high-energy motor vehicle accidents or combat-related explosions, have the potential to disrupt the native configuration of the knee and other joints. Among the most severe of these penetrating injuries are those that extend into the subchondral bone and result in pronounced deterioration of the articular surface. These injuries are commonly referred to as intra-articular fractures (IAFs).^1^ The presence of IAF significantly increases the risk of developing degenerative post-traumatic osteoarthritis (PTOA) by nearly 20-fold when compared to joint injuries that do not extend into the subchondral bone.^2^ Unlike idiopathic osteoarthritis, which typically stems from prolonged joint wear and tear, PTOA is characterized by rapid and progressive osteochondral deterioration and often affects younger individuals.^3^ Key factors contributing to the transition to PTOA after IAF injuries include the loss of articular cartilage, synovial damage, subchondral bone injury, and persistent joint incongruities.

Alongside these observed tissue changes, dysregulated inflammation significantly contributes to PTOA pathology by hindering osteochondral regeneration. Immediately after injury, immune cells flood the wounded area, causing substantial inflammation. Inflammatory cytokines, like IL1α, IL1β, and tumour necrosis factor α (TNFα), are secreted by infiltrating leucocytes.^4^ These cytokines activate local immune cells and promote chemotactic chemokine release, facilitating cell migration to the wound site. Post-IAF, this inflammatory phase is amplified and prolonged, disrupting normal osteoarticular healing. Prior research shows that inhibiting these cytokines using monoclonal antibodies or small molecule inhibitors, targeting IL1 specifically, may protect against PTOA.^5^ ANR blocks IL1α and IL1β cytokines’ activation of receptors produced by damaged tissues and infiltrating immune cells.^6^ By inhibiting IL1 signal transduction, ANR reduces intracellular cytokine transcription, controlling inflammation propagation. Clinically available ANR slows the progression of degenerative diseases like rheumatoid arthritis and degenerative OA.^5,7^ Recent reports indicate that ANR helps dampen the inflammatory response and protect against further damage but does not completely reverse the pathobiology of PTOA following IAF.^8^ Although anti-inflammatory agents mitigate additional damage, it is likely that preservation and/or restoration of injured tissue will require a multi-pronged approach, combining therapies for both inflammation mitigation and tissue regeneration.

Chondrogenic agents offer some promise in revitalizing articular cartilage.^9^ Kartogenin (KGN) and its structural analogue KA9 are small heterocyclic molecules with chondroinductive properties.^9^ KGN induces chondrogenic differentiation of mesenchymal stem cells through the JNK/RUNX pathway while suppressing the pro-osteogenic β-Catenin/RUNX2 pathway, promoting Collagen II and aggrecan transcription.^10^ While KGN has promoted cartilage regeneration in some animal models, its effects on more severe injuries like IAF remain unexplored, as does its combination with anti-inflammatory therapies such as ANR. Therefore, the objective of the current study was to assess the effects of KGN and KA9 in conjunction with ANR on PTOA progression following IAF in a small animal model. We hypothesize that this combined approach, addressing both inflammation and osteochondral damage, would enhance healing and reduce PTOA development after IAF. Based on this hypothesis, results from this study may be translated to the clinic as a potential treatment strategy for traumatic IAF leading to PTOA.

## Methods

### Animals

This study was approved by our local Institutional Animal Care and Use Committee and conducted in accordance with the Animal Welfare Act and the principles outlined in the Guide for the Care and Use of Laboratory Animals.^11^ An ARRIVE checklist is included to demonstrate adherence to the ARRIVE guidelines (Supplementary Material). Skeletally mature male Lewis rats (300 to 350 g) were obtained from Charles River Labs (USA) and housed according to standard animal husbandry practices with unrestricted access to food and water. The study was limited to male rats, a methodological decision consistent with historical trends in combat casualty care research, which has predominantly focused on male subjects owing to their overwhelming representation in military personnel, and the disproportionately higher incidence of combat-related trauma within this demographic. Body weights (BW) were continuously monitored throughout the experimental period to ensure animal welfare.

### Drug preparation

Osmotic pumps (Alzet; DURECT, USA) were filled with 0.9% physiological saline (vehicle controls) or ANR (Kineret; Sobi Biopharmaceuticals, Sweden) prepared to a working concentration of 24 µg/ml in saline and primed overnight at 37°C prior to implantation. Additionally, working stocks of KGN (MilliporeSigma, USA) and its chemical analogue KA9 (Scripps Research Institute, USA) were prepared by diluting to a final working concentration of 1 µg/100 µl in saline. Injectables were aseptically prepared the day of injection and were kept under sterile conditions.

### Intra-articular fracture model and drug delivery

Rats were anesthetized using inhaled isoflurane in oxygen ranging from 1% to 3% and administered a singular dose of slow-release buprenorphine at 1 mg/kg body weight for analgesia. They then underwent baseline µCT scanning before a controlled blunt impact (5 joules) to their left hindlimb to induce IAF.^12^ Scans were conducted after injury to confirm IAF. Animals that did not develop fracture after injury were excluded from future analysis. Animals were block randomized to time and treatment and assigned treatment groups defined as; Saline Pump + Saline Injection (Sal/Sal), Saline Pump + KNG (Sal/KGN), Saline Pump + KA9 (Sal/KA9), ANR Pump + KGN (ANR/KGN), and ANR Pump + KA9 (ANR/KA9). Subsequently, osmotic pumps, with payloads (e.g. saline or ANR) tailored to each experimental group, were surgically implanted. Over the next 14 days, saline or ANR was continuously infused via the osmotic pumps until their eventual removal. After implantation rats received a 100 µl injection of either saline, KGN, or KA9, based on their experimental group. Intra-articular injections were administered weekly for six weeks.

### Tissue, blood, and synovial fluid procurement

Whole blood was collected from the tail artery before, immediately after, and at 14 and 56 days post-injury. After blood collection, serum was obtained through centrifugation at 1,000 RPM for ten minutes at room temperature. Following euthanasia using a sodium pentobarbital overdose (I.C. injection, EUTHASOL; Virbac AH, Inc., USA) at the specified study endpoints (14 or 56 days), synovial fluid (SF) was extracted from the hindlimb knees by placing folded Whatman paper pieces into the joint cavity to absorb the SF. These samples were then rapidly frozen in liquid nitrogen for later processing and analysis. Subsequently, the entire hindlimbs were excised and preserved in 10% formalin for subsequent ex vivo imaging and histological examination. Additionally, complete livers and kidneys were harvested and weighed to assess systemic toxicity.

### In vivo micro-CT and fracture assessment

To confirm the presence of IAF and assess their severity, micro-CT (µCT) scans were conducted both before and after impact, using a voxel size of 50 µm, a 1 mm aluminium filter, a voltage of 65 kV, a current of 770 uA, and an exposure time of 56 ms with a 360° rotation in 0.3° increments (SkyScan 1278; Bruker Corporation, USA). 3D images were generated from the reconstructed scans, and grey values were adjusted for visualization and semi-quantitative evaluation of the injured limbs according to the AO/OTA Fracture Classification and Dislocation Compendium.^13^ Reviewers were blinded to sample metadata, and images were scored based on the location of the fracture and level of intensity. Frequency of fractures was noted for both the femur and tibia from each sample, and tallied.

### Contrast enhanced micro-CT

Cartilage thickness and composition were analyzed using contrast-enhanced µCT (CE-µCT). Tibial heads were immersed in 1 ml of 12 mg/ml CA4+ solution (a positively charged iodated contrast agent) for 24 hours at 4°C.^11^ After incubation, the tibial heads were blotted dry and scanned at 70kV and 112 uA with an integration time of 300 ms using a 0.5 mm aluminium filter and a voxel size of 9 micrometers (1172 SkyScan system, Bruker Corporation). CTan (Bruker Corporation) processed the reconstructions, and DataViewer (Bruker Corporation) aligned them. Regions of interest (ROIs) were defined to separate cortical and trabecular structures while excluding soft-tissue. These ROIs were refined, and bone mineral density and bone morphometry (BMM) were measured using predefined software parameters. BMM measurements included: total or tissue volume (TV), bone volume (BV), BV/TV, bone surface (BS), degree of aniostropy (DA), fractal dimension (FD), trabecular number (TbN), Tb thickness (TbTh), Tb separation (TbSp), and connectivity density (ConnD). Object number (ObN) CA4+-enhanced images were also analyzed to extract comprehensive data on cartilage thickness and CA4+ staining density across different injury and treatment groups.

### Soluble factor analysis

Snap-frozen SF samples on Whatman paper were rehydrated in 300µL of lysis buffer (Catalogue # EPX-99999-000, Thermo Fisher Scientific, USA) overnight at 4°C with gentle agitation. The resulting extracts were then divided into aliquots for use in downstream assays. Serum samples and SF extracts were subjected to analysis to detect soluble osteochondral degradation markers, specifically COMP (Catalogue # MBS2512987; MyBioSource, Inc., USA), NTX1 (Catalogue # MBS454100; MyBioSource, Inc.), CTXI (Catalogue # MBS2024328; MyBioSource, Inc.), and CTXII (Catalogue # MBS2880519; MyBioSource, Inc.), following the manufacturer’s recommended protocols. Absorbance readings were recorded at 450 nm, and the resulting values were calculated in relation to the provided linear standards. Additionally, diluted SF samples were analyzed for soluble factors using a pre-designed multiplex immunoassay designed specifically for rat cytokines and chemokines (Catalogue # EPX220-30122-901; Thermo Fisher Scientific). In the case of synovial fluid samples, urea levels were measured via ELISA (Catalogue # MBS2600001; MyBioSource, Inc.) for data normalization.^14^ Any measurements that fell below the detection threshold were assigned a value equal to half of the limit of detection.

### Histological analysis

The hindlimb knee joints were fixed in 10% neutral buffered formalin for seven days, followed by saline rinsing and a ten-day decalcification using a 10% EDTA solution. Afterward, samples were processed, paraffin-embedded longitudinally, and sectioned to 5 micron thickness. These sections were then deparaffinized and stained with haematoxylin and eosin (H&E) and Safranin-O with Fast Green. For immunohistochemistry (IHC), sections were probed for CD68 (1:200, Bio-Rad Laboratories, Inc., UK), inducible nitric oxide synthase (iNOS) (1:200, Abcam, UK) on Day 14, DCSTAMP (1:50, Abcam) on Days 14 and 56 to assess osteoclast activity, and collagen II composition (1:200, Abcam) on Day 56. Brightfield images were captured using a Zeiss Axio Scan.Z1 and stitched together to create a composite image representing the knee’s cross-section. Evaluations were conducted by investigators blinded to treatment groups. Qualitative assessments of morphology and composition were performed by examining n ≥ 6 per group. Quantitative evaluation of PTOA manifestation followed the Osteoarthritis Research Society International (OARSI) guidelines.^15^ IHC analysis qualitatively assessed punctate staining in each section and compared it to control tissues.

### Statistical analysis

Data is reported as the mean (standard error of the mean (SEM)) with statistical analysis was performed by analysis of variance (ANOVA) with Šidák corrections for multiple comparisons, reported as the mean (SEM) with a significance level set at *α* = 0.05 using GraphPad Prism Software (GraphPad Software, USA). Sample sizes were determined by a fixed-effects ANOVA based on CT scans of cartilage thickness, where a number of six to eight per group and timepoint allowed for a power level of 0.8 and an α of 0.05, for a total of 76 animals on study.

## Results

### Fracture characteristic and physiological assessments

**Fig. 1:**
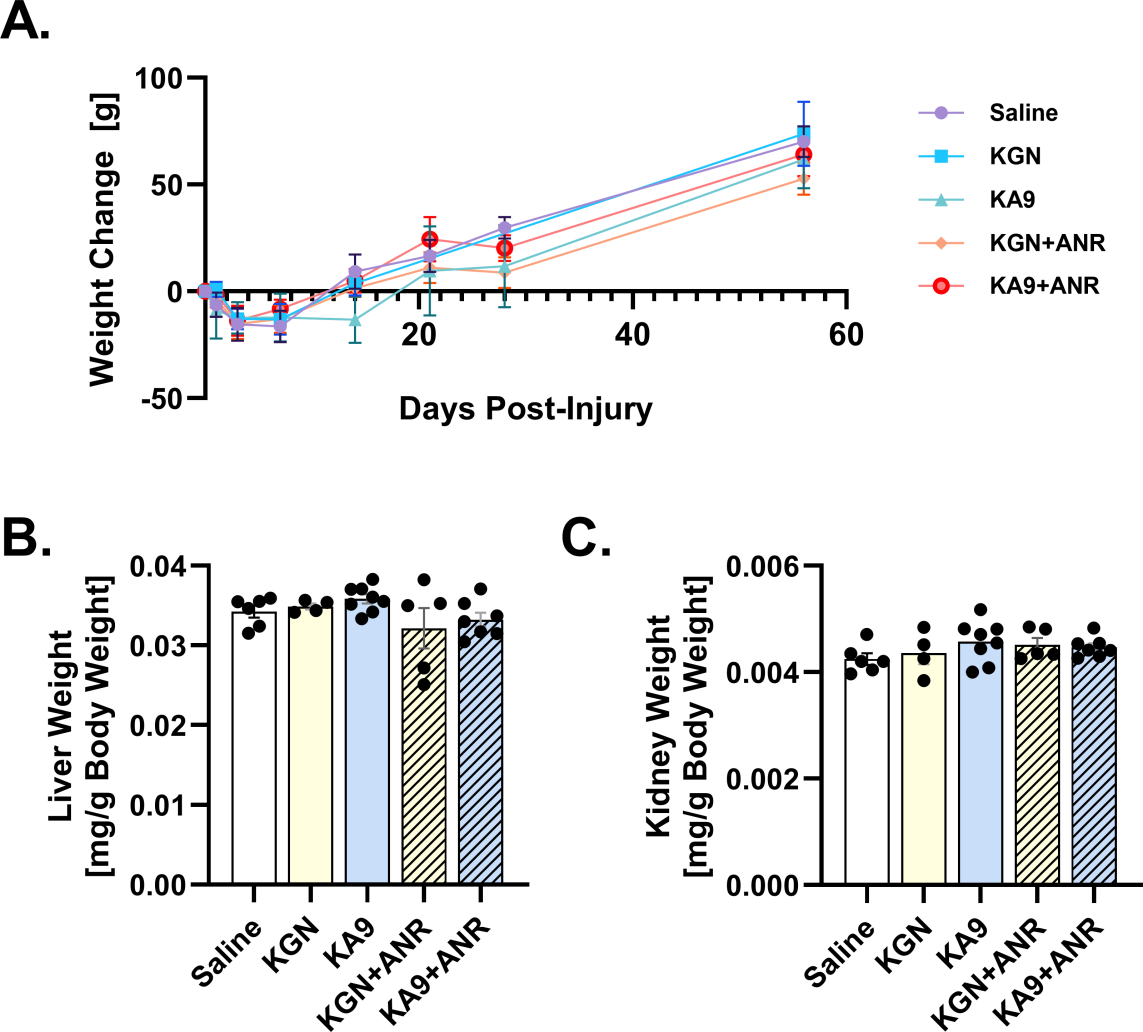
Systemic impact of immunoregenerative treatments on body and organ weights after intra-articular fracture. a) Body weight changes after intra-articular fracture. b) Liver weight normalized to body weight and c) kidney weight normalized to body weight on Day 14 post-injury. ANR, anakinra; KGN, kartogenin.

Tibial fractures consistently exhibited a multifragmentary nature, and were classified as either partial-articular (41B) or full articular fractures (41C) in terms of AO/OTA classification or severity. Analysis of body weights revealed a consistent pattern where all animals initially experienced weight loss, followed by subsequent weight gain (Figure 1a). This pattern suggests that the interventions did not have an adverse impact on overall animal wellbeing. Furthermore, in addition to monitoring changes in body weight, we conducted assessments of liver (Figure 1b) and kidney (Figure 1c) weights following the maximum treatment exposure on Day 14. These measurements were then compared to the control group, and the analysis revealed no differences in liver or kidney weights between the groups, even after normalizing the values to body weight.

### Immunoregenerative approach modulates systemic inflammatory profile and cytokine response

**Fig. 2:**
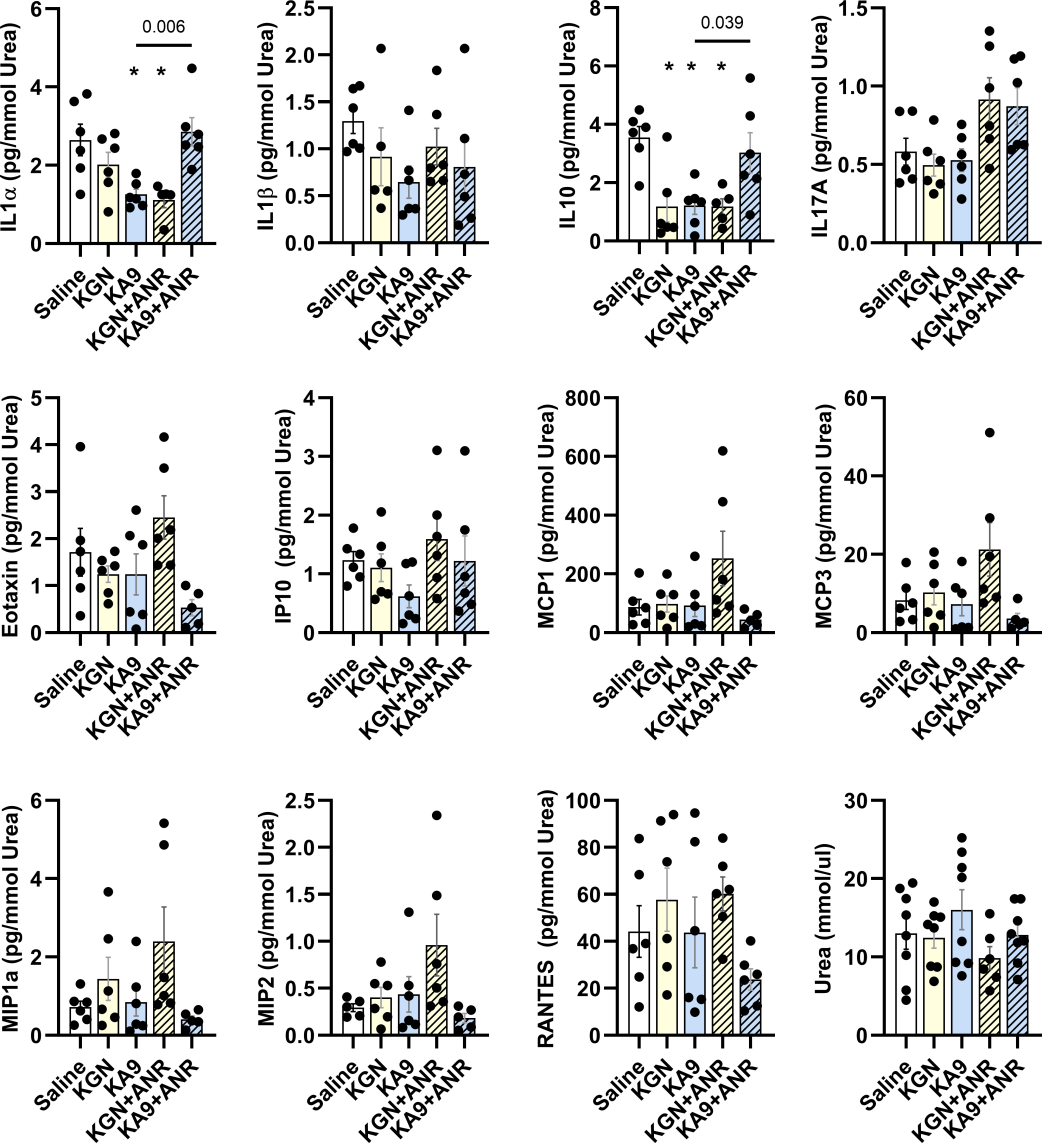
Influence of immunoregenerative treatments on local inflammatory factor expression after intra-articular fracture. We examined the levels of inflammatory cytokines (IL1α, IL1β, IL10, IL17A) and chemokines (Eotaxin, IP10, MCP1, MCP3, MIP1α, MIP2, and RANTES) in synovial fluid using multiplex immunoassays at the Day 14 endpoint. These results were standardized by urea content and are reported as pg/mmol. Urea levels were determined by enzyme-linked immunosorbent assay and expressed as mmol/μl. Each data point represents an individual sample (n ≥ 6 animals). The means are presented with standard error of the mean (SEM), and individual p-values are reported for each analytical test. *p < 0.05 compared with saline control, using analysis of variance with Šidák corrections for multiple comparisons. ANR, anakinra; KGN, kartogenin.

No discernible variations in the expression levels of the following cytokines were detected in the SF across the experimental groups (Figure 2): IL1β, IL17A, Eotaxin (CCL11), IP10 (CXCL10), MCP1 (CCL2), MCP3 (CCL8), MIp1a (CCL3), MIP2 (CXCL2), and RANTES (CCL5). Conversely, both the KA9 and KGN+ANR treatments exhibited a substantial reduction of nearly 60% in IL1α levels when compared to the saline controls (both p = 0.011). Notably, the KA9+ANR treatment effectively reversed the IL1α reduction initiated by KA9, restoring IL1α expression to levels comparable to those of the saline control (p = 0.006). As for IL10 expression, our results indicated that treatment with KGN (p = 0.022), KA9 (p = 0.003), and KGN+ANR (p = 0.003) each led to a reduction of over 50% relative to the saline controls. However, the KA9+ANR treatment reversed this reduction, reinstating IL10 expression to a level comparable to the saline control, and nearly twofold higher than that of KA9 (p = 0.039).

### Immunoregenerative approach modulates osteochondral degradation marker expression

**Fig. 3:**
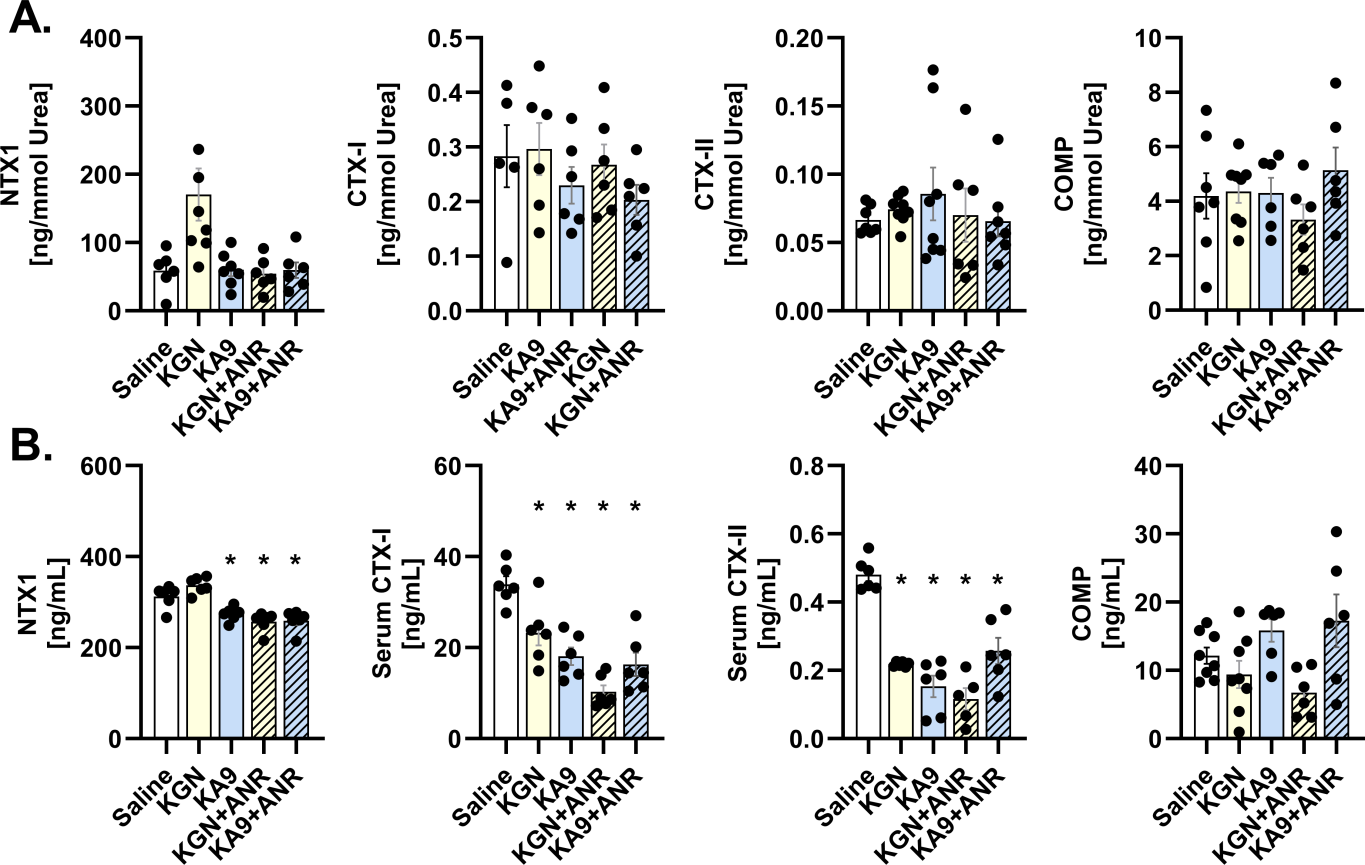
Effects of immunoregenerative treatments on local and systemic osteochondral breakdown expression following intra-articular fracture. a) Local expression of osteochondral markers in synovial fluid samples collected on Day 56, normalized to urea content. b) Systemic expression of osteochondral markers in serum samples collected on Day 56. Each data point represents an individual sample (n ≥ 6 animals). The means are presented with standard error of the mean (SEM), and individual p-values are reported for each analytical test. *p < 0.05 compared with saline control, using analysis of variance with Šidák corrections for multiple comparisons. ANR, anakinra; KGN, kartogenin.

There were no discernible differences in osteochondral degradation markers among the groups in the SF collected 56 days after injury (Figure 3a). However, in contrast to these findings, the serum results (Figure 3b) indicated a modulation of osteochondral breakdown through immunoregenerative treatment. KGN + ANR (p = 0.002), KA9 (p = 0.011), and KA9 + ANR (p = 0.003) treatments each reduced NTX1 expression relative to saline controls. Furthermore, KGN + ANR further reduced NTX1 expression relative to KGN (p < 0.001). CTXI expression was reduced by over 50% in KGN (p = 0.010), KA9 (p < 0.001), KGN+ANR (p < 0.001), and KA9+ANR (p < 0.001) compared to the saline control, with the KGN + ANR treatment displaying a greater reduction in CTX-I expression than KGN alone (p < 0.001). CTXII expression also revealed a 50% or greater reduction in expression in all treatment groups compared to saline controls (p < 0.001 each). Addition of ANR to KGN further reduced this expression relative to its control, KGN (p = 0.007). Conversely, compared to its control (Sal/KA9), ANR + KA9 treatment resulted in increased CTXII expression (p = 0.011), although still expressing below saline’s levels. Finally, bone breakdown marker COMP was not shown to be affected by any treatment compared to control.

### Immunoregenerative approach augments bone morphometry and bone mineral density

**Fig. 4:**
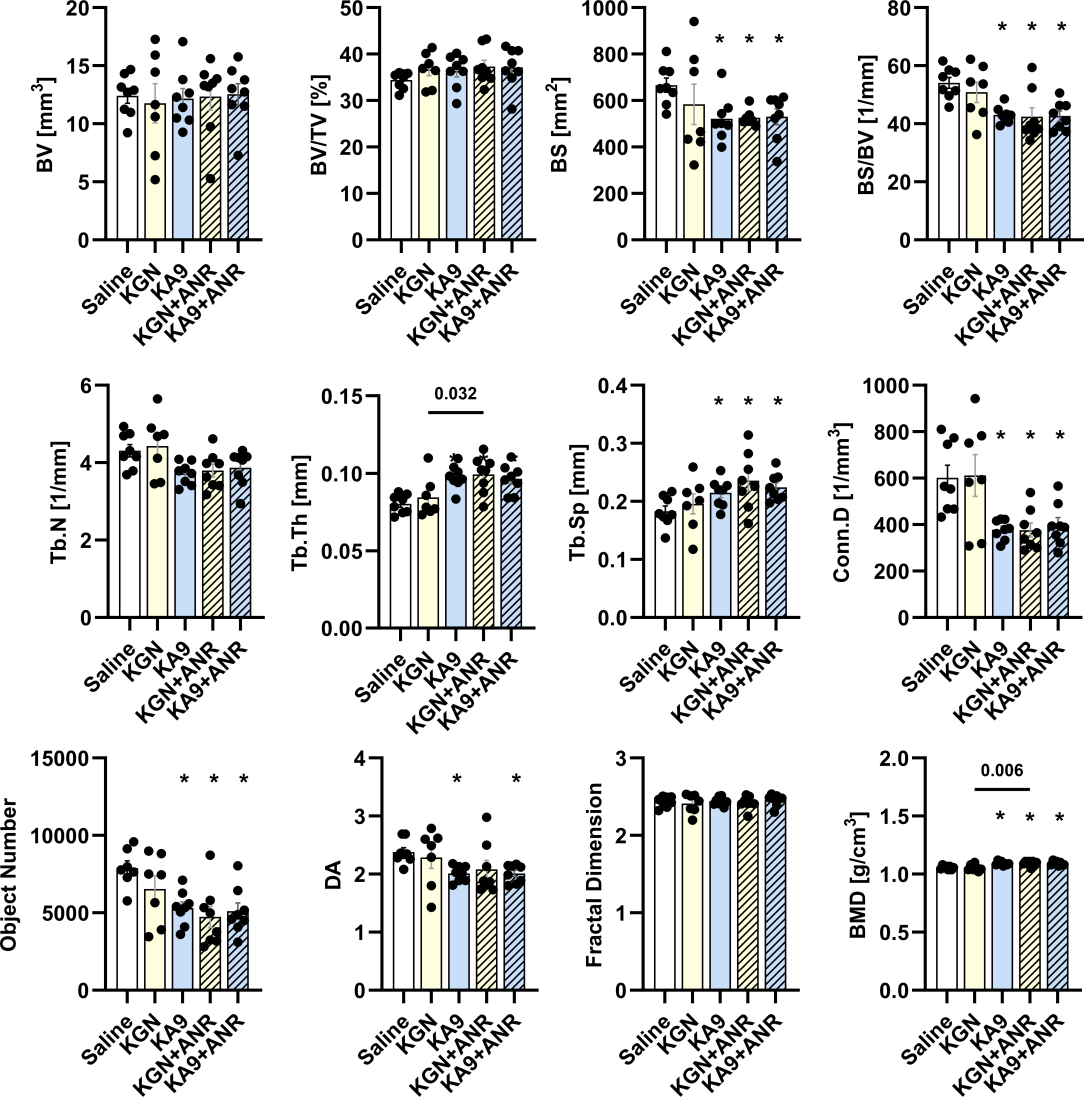
Impact of immunoregenerative treatments on morphometry and bone mineral density after intra-articular fracture. Bone morphometric measurements (BMM) were derived from ex vivo scans of the tibial plateau on Day 56. These measurements encompass bone volume (BV), BV to total volume ratio (BV/TV), bone surface (BS), BS/BV ratio, trabecular number (Tb.N), trabecular thickness (Tb.Th), trabecular separation (Tb.S), connectivity density (Conn.D), object number (Obj.N), degree of anisotropy (DA), and fractal dimension (FD). Additionally, bone mineral density (BMD) was calculated from the tibial plateau scans and is expressed as g/cm^3^. Each data point represents an individual sample (n ≥ 6 animals). The means are presented with standard error of the mean (SEM), and individual p-values are reported for each analytical test. *p < 0.05 compared with saline control, using analysis of variance with Šidák corrections for multiple comparisons. ANR, anakinra; KGN, kartogenin.

Quantitative ex vivo µCT results (Figure 4) reveal a lack of discernible differences in BMM among the groups with respect to parameters such as BV, BV/TV, Tb.N, and FD. In contrast, the data demonstrate that KA9, KGN+ANR, and KA9+ANR treatments each led to an approximately 25% reduction in BS when compared to saline controls (p = 0.010, p = 0.007, and p = 0.011, respectively). Similarly, KA9, KGN+ANR, and KA9+ANR also resulted in a decreased BS/BV ratio in comparison to the saline controls (p < 0.001, p = 0.001, and p < 0.001, respectively). Regarding bone structure, KGN, KA9, and KA9+ANR treatments were found to increase Tb.Th in comparison to the saline controls. Notably, KA9+ANR exhibited an even more significant increase in Tb.Th when compared to KA9 alone (p = 0.032). Tb.S was also affected by the treatments, with KA9, KA9+ANR, and KGN+ANR all displaying increases relative to the saline controls (p = 0.042, p = 0.042, and p = 0.027, respectively). Furthermore, both individual treatments (KGN and KA9) and combined treatments (KGN+ANR and KA9+ANR) were found to reduce Conn.D and Obj.N when compared to the saline controls (p < 0.010 each). Besides bone morphometric factors, analysis of bone mineral density (BMD) revealed that compared to saline treatment, KA9 (p < 0.001), KGN+ANR (p = 0.001), and KA9+ANR (p < 0.001) all increased BMD. Comparing within groups, KGN+ANR expressed higher BMD than KGN (p = 0.006).

### Immunoregenerative approach augments cartilage thickness and composition

**Fig. 5:**
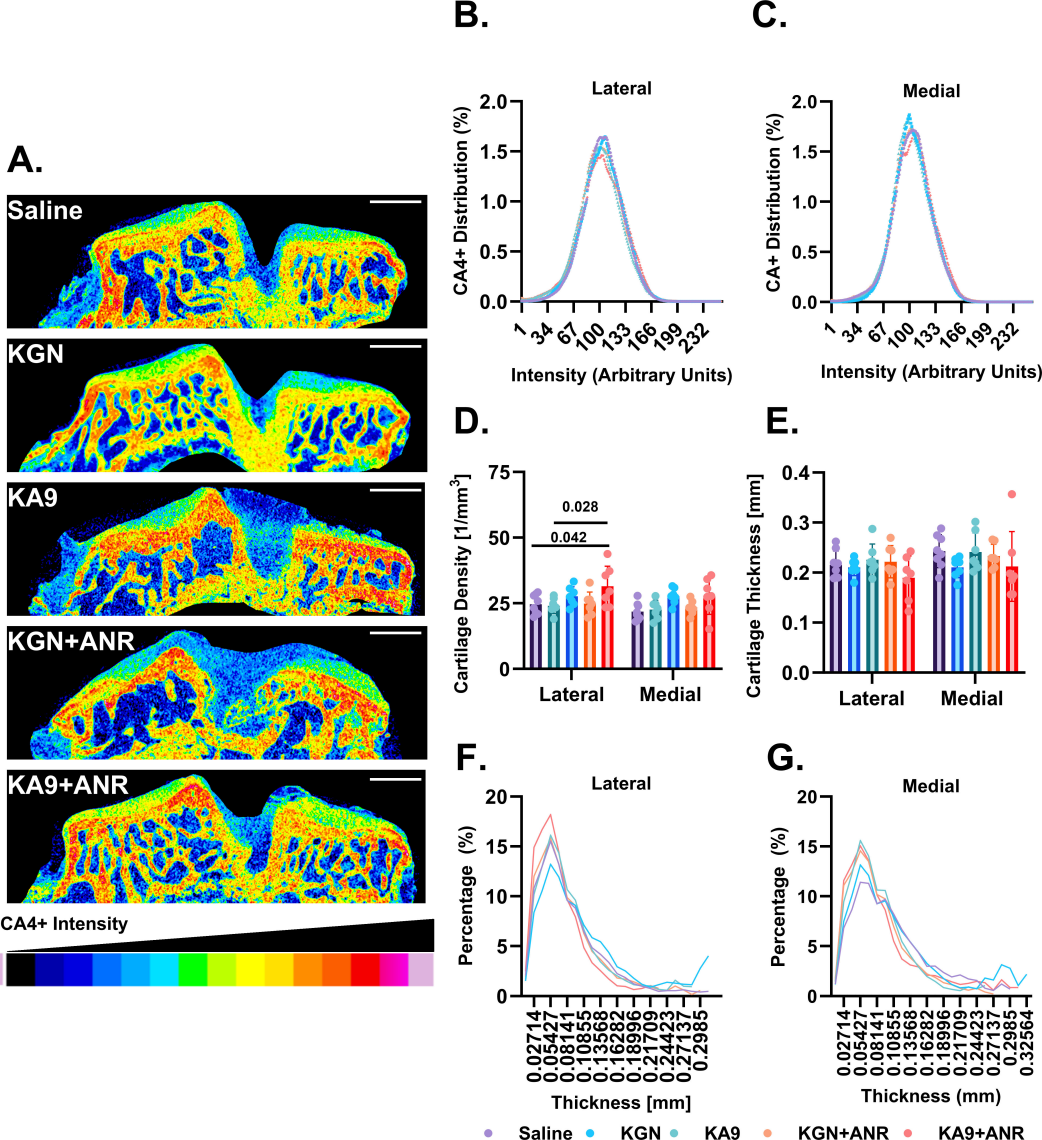
Effect of immunoregenerative treatments on articular cartilage thickness and composition. a) Representative microCT (μCT) images using CA4+ as a contrast agent are shown for each experimental group at 56 days post-injury, with the colour scale indicating stain intensity (scale bar: 1 mm). Histograms depict the distribution of articular surface thickness and CA4+ density across the entire b) and d) lateral and c) and e) medial tibial epiphysis, respectively. Summary data are presented illustrating f) CA4+ density and g) mean cartilage thickness for each group. Each data point represents an individual sample (n ≥ 6 animals). The means are displayed with standard error of the mean (SEM), and individual p-values are provided for each analysis in the main text. ANR, anakinra; KGN, kartogenin.

CE-µCT analysis revealed variability in cartilage staining intensity, and stain distribution across the articular surface (Figure 5a). CA4+ staining intensity exhibited a relatively normal distribution over the density range between groups, with slight variations in peak height and width (Figures 5b and 5c). Summation of cartilage density across the ROI reveals differences between groups (Figure 5d). On the lateral aspect, dual KA9 + ANR treatment increased overall density relative to saline controls (p = 0.004), with no other treatment differing from saline controls. KA9 + ANR also increased overall density compared to KGN and KGN + ANR (p = 0.002 and p = 0.008, respectively). On the medial aspect, density summation data again showed that dual KA9 + ANR treatment increased CA4+ relative to saline controls (p = 0.013), while KA9 treatment also increased CA4+ intensity relative to saline controls (p = 0.014).

While no differences in the maximum cartilage thickness (Figure 5e) were observed between treatment groups for both the lateral and medial aspects of the tibial articular surface*,* distribution analyses (Figures 5f to 5g) revealed an increase in the overall peak height with most treatments compared to controls in both the medial and lateral aspects, representing a rightward shift in cartilage thickness distribution in response to treatments compared to controls. Most treatments, except for medial KGN + ANR, exhibited a defined right shift and thus increased cartilage thickness compared to controls.

### Immunoregenerative approach augments histopathological presentation of tibia

**Fig. 6:**
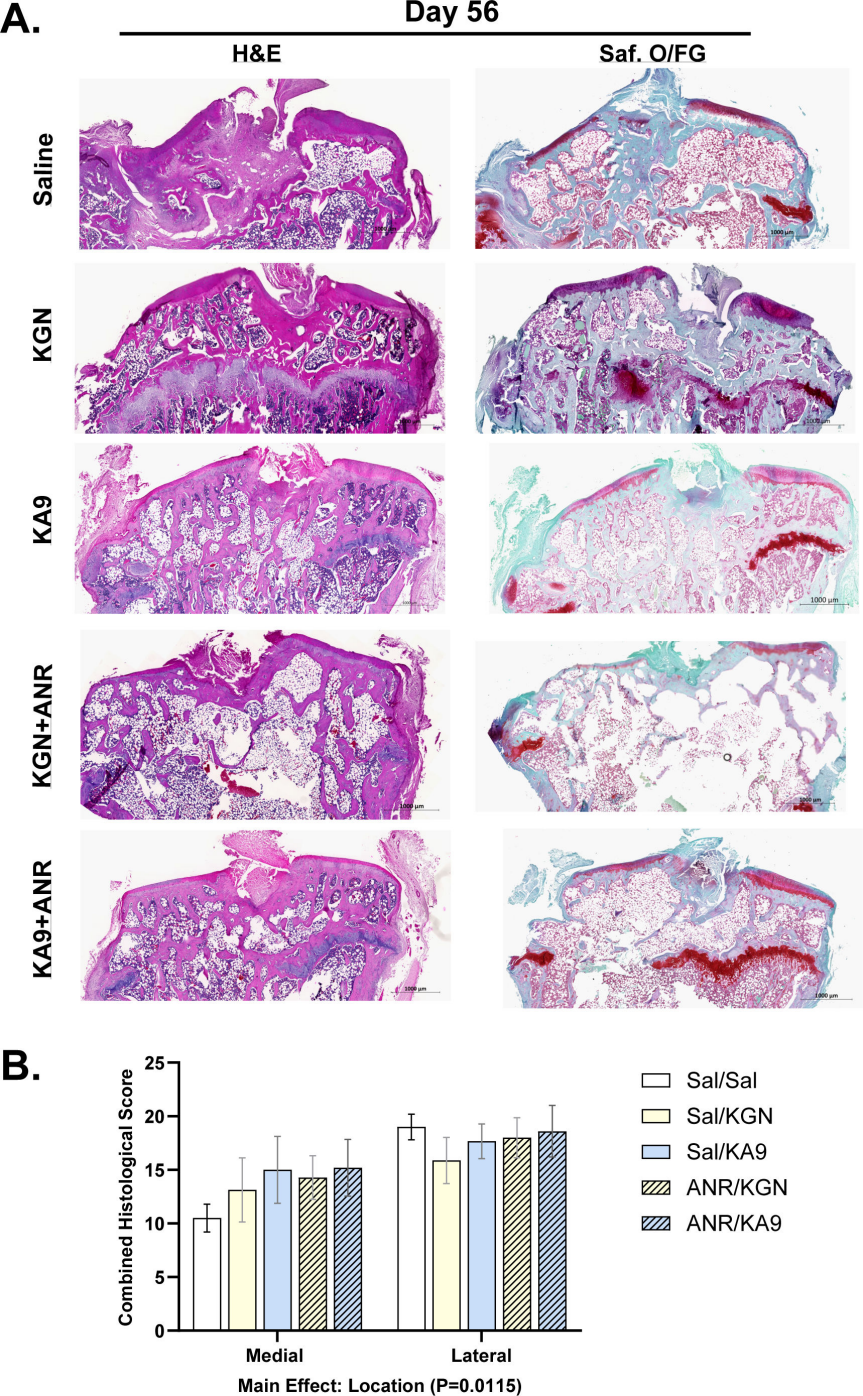
Impact of immunoregenerative treatments on chronic histopathological presentation of the tibia. a) Representative micrographs displaying haematoxylin and eosin and Safranin-O staining of both the medial and lateral aspects of the tibial epiphysis. Scale bars are set to 1,000 μm. b) Osteoarthritis Research Society International modified Mankin scores, obtained separately for the medial and lateral aspects, for each group. Each data point represents the score from an individual sample (n = 4 to 6 animals). The means are presented with standard error of the mean, and individual p-values are reported for each analysis in the main text. ANR, anakinra; KGN, kartogenin.

Representative H&E and Safranin-O staining of the medial and lateral aspects of the tibial epiphysis at 14 (Supplementary Figure a) and 56 days post-injury (Figure 6) show similarities in cellular infiltration and cartilage staining between experimental groups. Accordingly, quantitative scoring of the 56 day micrographs via the modified OARSI score revealed worse pathology on the lateral aspect of the tibial epiphysis than the medial across all groups (main effect, p = 0.007), but that no meaningful differences exist between experimental groups.

**Fig. 7:**
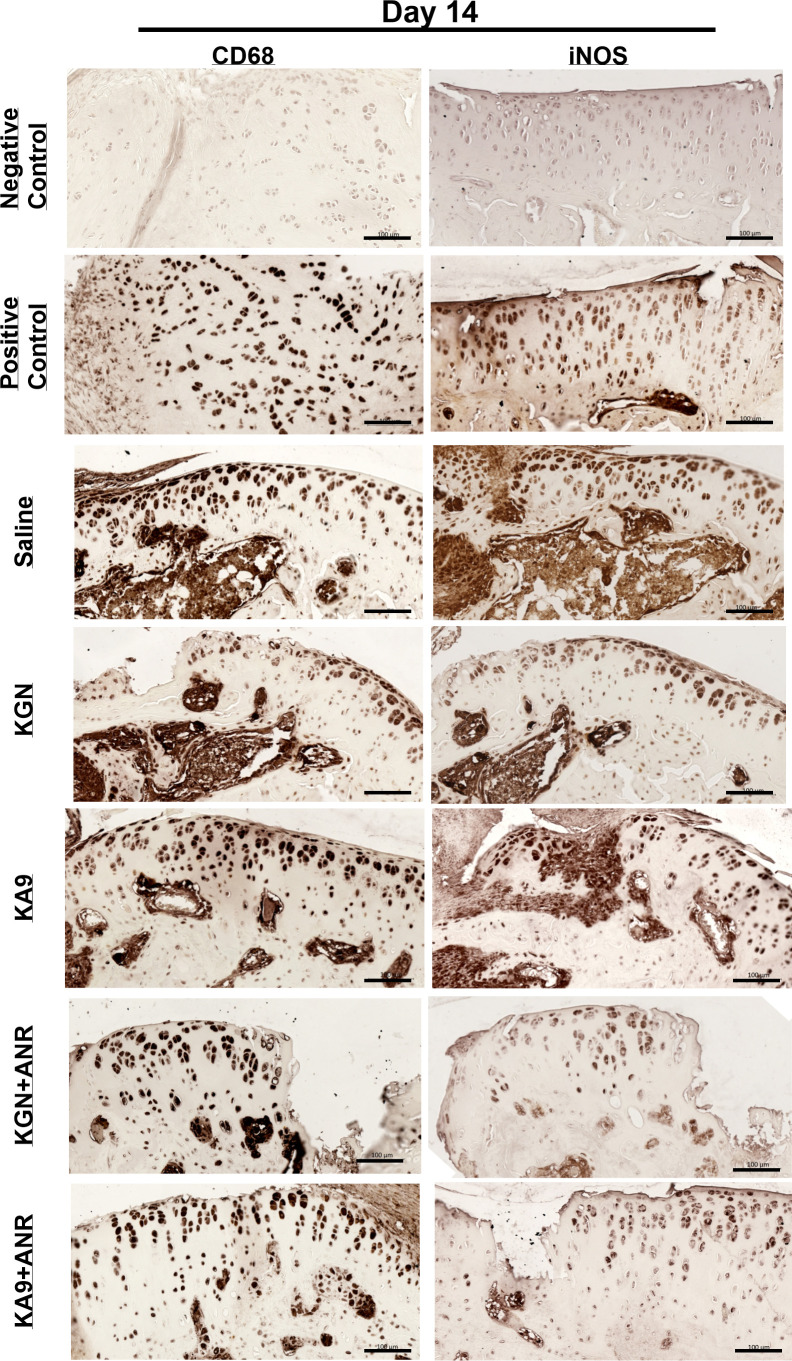
Effects of immunoregenerative treatments on early inflammation and bone degradation. Representative images from each group at Day 14 post-injury as well as positive and negative staining controls. The left side of the figure includes representative tissues from each group stained for the pan macrophage marker, CD68. The right side of the figure includes representative tissues from each group stained for inflammatory marker inducible nitric oxide synthase (iNOS). Each image represents a region of interest at or adjacent to the major site of injury at either the lateral or medial aspect of the tibial head. Scale bars are set at 100 μm for reference. ANR, anakinra; KGN, kartogenin.

Tissues collected at 14 days post-injury were stained for DCSTAMP, CD68, and iNOS to assess osteoclast activity, macrophage infiltration, and pro-inflammatory activity (Figure 7). Saline controls displayed significant osteoclast activity in both the lateral and medial aspects of the articular surface tibial epiphysis. KA9, KA9 + ANR, and KGN + ANR groups showed slightly higher DCSTAMP staining than saline controls in both medial and lateral aspects of the tibial epiphysis. Likewise, CD68 staining revealed robust macrophage infiltration across the articular surface and within all groups, with KA9, KA9 + ANR, and KGN + ANR-treated groups showing greater infiltration than saline controls. Finally, iNOS staining was observed in all groups, with KA9 and KA9 + ANR displaying the most intense staining.

**Fig. 8:**
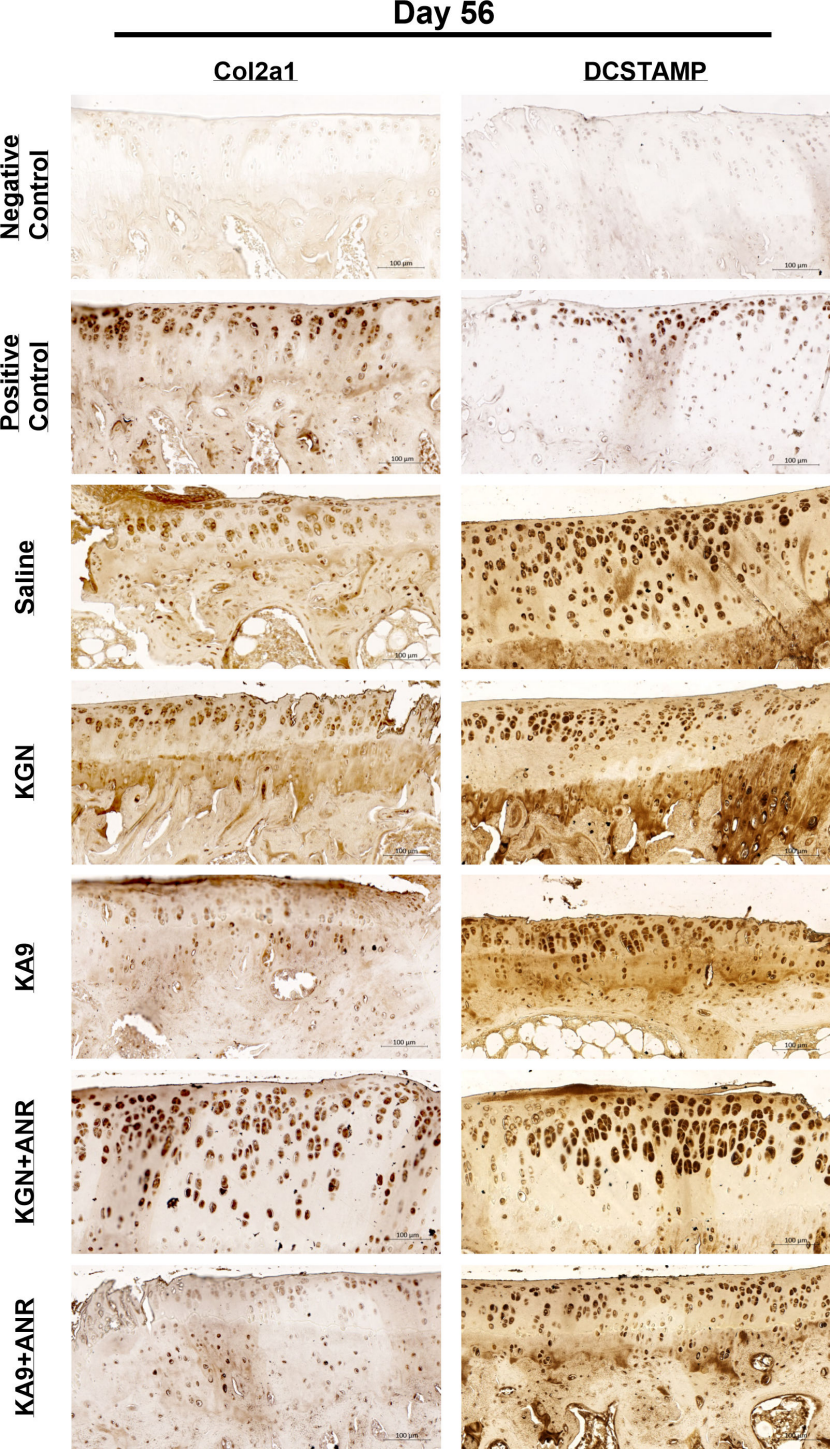
Effects of treatments on chronic immune cell infiltration and bone and cartilage degradation. Representative images from each group at 56 days post-injury are showcased, as well as positive and negative staining controls. The left side of the figure includes representative tissues from each group and stained collagen marker, collagen type II alpha I chain (Col2a1). The right side of the figure includes representative tissues from each group and stained for osteoclast/bone degradation marker, DCSTAMP. Each image represents a region of interest at or adjacent to the major site of injury at either the lateral or medial aspect of the tibial head. Scale bars are set at 100 μm for reference. ANR, anakinra; KGN, kartogenin.

Representative images of Day 56 harvested tissues were stained for osteoclast activity using DCSTAMP, pan-macrophage infiltration using CD68, and collagen preservation via collagen II (Figure 8). Upon revisiting osteoclast activity through DCSTAMP at the Day 56 timepoint, our staining results displayed a robust expression in all groups, both on the medial and lateral surfaces. When comparing the groups in terms of staining intensity and quantity, it was evident that KA9, KGN, and KGN + ANR exhibited the most pronounced staining, surpassing that of saline. KA9 + ANR staining resembled that of the saline group. Revisiting the pan-macrophage infiltration, as indicated by CD68, all tissues continued to exhibit positive CD68 expression, signifying ongoing immune cell infiltration and active inflammation. When comparing the groups, the Saline group exhibited the most pronounced CD68 expression compared to all other groups, with no notable differences observed among the treatment groups. Finally, with regards to collagen reformation assessed through collagen II IHC staining, our results once again demonstrated positive staining in all tissues. When comparing the groups, saline, KA9, and KGN all exhibited more robust collagen II expression compared to each of the dual treatment groups.

## Discussion

The primary aim of this study was to investigate the impact of chondrogenic agents, specifically KGN and its structural analogue KA9, in mitigating PTOA following IAF. We examined the compounds both as individual treatments and in combination with ANR, hypothesizing that ANR’s anti-inflammatory properties might create a more favourable environment for chondroprotective agents to potentially attenuate PTOA development. Our decision to include ANR in this study was based on our previous research, where we observed that the anti-inflammatory effects of ANR partially mitigated the progression of PTOA induced by IAF compared to control groups. In that prior study, ANR was effective in reducing pro-inflammatory factors (IL1α and IL17), osteochondral breakdown markers (CTXII, COMP, and TRAP), and increasing the osteogenic marker ALP. Furthermore, ANR demonstrated positive effects on bone volume and other morphometric markers.^8^ While these results showed promise, they did not fully prevent PTOA, emphasizing the importance of a comprehensive therapeutic approach and motivating our investigation of KGN and KA9.

Our research findings provide some support for our hypothesis, suggesting that both KGN and KA9 may offer some protection against PTOA development, and that this effect may be enhanced when combined with ANR. It is important to note that the effects were not consistent across all measured outcomes. The most notable findings in our study relate to the impact of our immunoregenerative approach on cartilage density as assessed by CE-µCT using CA4+ as our contrast agent.^16-19^ CA4+ covalently bonds with sulfated glycosaminoglycans (sGAG), establishing a direct correlation between CA4+ staining intensity and sGAG density.^20^ Using this technique, we observed a relatively normal distribution of CA4+ staining across the articular surface (Figures 5a to 5c). Compared to saline controls, cartilage density appeared to be increased in the medial aspect of the tibial epiphysis in the KA9-treated group, and in both the medial and lateral aspects in the KA9 + ANR group (Figure 5d). However, it is crucial to acknowledge that these increases were not observed in all treatment groups. No differences in maximum cartilage thickness were observed with any of the treatments (Figures 5e to 5g).

Beyond our imaging outcomes, direct measurement of markers of osteochondral remodelling is vital for assessing disease progression and evaluating the impact of disease-modifying arthritis drugs on PTOA. Numerous working groups and consortiums have dedicated significant effort to validate soluble biomarkers in serum or urine, including CTX-I, CTX-II, NTX-I, collagen II, and COMP, to serve as reliable prognostic indicators for osteoarthritis, encompassing both PTOA and OA.^21,22^ We assessed the effects of our treatments on such markers in both the synovial fluid (SF) and serum, finding that both KGN and KA9 reduced serological levels of NTX1, CTX-I, and CTX-II in alignment with a prior report,^23^ and that these protective effects were enhanced by concurrent administration of ANR. Similarly, we examined synovial fluid cytokine composition at 14 days post-injury, corresponding with the conclusion of ANR treatment and pump removal. At this timepoint, IL1α and IL10 exhibited differential expression in treated versus control groups. IL1α expression was reduced with KA9 and KGN + ANR treatment, while IL10 expression was decreased by treatment with KGN, KA9, and KA9 + ANR.

These outcomes present a mixed picture when compared with the existing literature. Reduced IL1α expression via KGN + ANR is generally consistent with expectations, as IL1α and IL1β are targets of ANR.^24^ The IL10 result, however, contrasts with a prior study showing that KGN treatment induces IL10 production in concert with PTOA mitigation.^25^ One possible explanation for this discrepancy is the model used. The aforementioned study used a murine monosodium iodoacetate (MIA) model to induce PTOA, while our study focused on induced trauma in a rat. It is plausible that the dynamics of IL10 production are different either due to species-related factors or disparities between chemically based models, which primarily impact the soft-tissue, and severe injury models, which involve a fracture healing component.

With regard to other similar models, Furman et al^26^ evaluated the effect of IL-1 inhibition therapy in a mouse PTOA model. In that study, they also found that use of IL-1RA helped to slow the progression of PTOA through a reduction in biomarkers associated with inflammation and osteochondral breakdown. As in our study, they found that serum CTX-1 decreased with local IL-1RA administration. However, while we found a statistically significant reduction in serum CTX-II in all treatments, their study did not see this effect. Comparing our results to theirs, it is evident that the reductions in CTX-1 and CTX-II we observe in serum may be due primarily to the addition of prochondrogenic factors (i.e. KGN and KA9), and not necessarily inhibition of IL-1RA. Moreover, while Furman et al^26^ saw a reduction in local COMP that was absent in our prior results,^8^ we observed a similar reduction when using prochondrogenic factors, regardless of concurrent ANR administration. These differences are important to acknowledge, and may stem from species variations or differences in model selection which warrant further investigation.

On the topic of model selection, one potential limitation of our study is the severity of the fractures we induced. While our data support the notion that immunoregenerative treatment after IAF may offer some protective benefit against the progression of PTOA, we acknowledge that the complex pathobiology of these severe fractures may mask some of the benefits of this approach for less severe joint injuries that also lead to PTOA. Further evaluation of these therapies, particularly KGN + ANR and KA9 + ANR, in alternative PTOA models (e.g., indentation, meniscal resection, ACL transection) may help to discern indications where the clinical impact of this approach is more apparent or meaningful to the target patient population.^27^ In fact, Mohan et al^23^ used an ACL transection model to look at the effects of Kartogenin on OA progression. There, they found that KGN decreased COMP and CTX-1 levels compared to controls. While interesting and in support of the use of KGN, this model does not fully replicate the type of injuries endured by active military Service members.

Our findings support the idea that immunoregenerative treatments combining chondrogenic and anti-inflammatory compounds may offer protection against PTOA development by preserving sulfated glycosaminoglycan density. This finding is consistent with another study using similar approaches (e.g., KGN with NSAIDs).^28^ This convergence of outcomes suggests that further research into immunoregenerative treatment for PTOA in additional models, including higher-order species, is clearly warranted to optimize treatment strategies, better define which specific aspects of PTOA progression these treatments can effectively address, and more fully evaluate its potential for clinical translation.

## Data Availability

The data that support the findings for this study are available to other researchers from the corresponding author upon reasonable request.
